# A New Soft Computing Method for K-Harmonic Means Clustering

**DOI:** 10.1371/journal.pone.0164754

**Published:** 2016-11-15

**Authors:** Wei-Chang Yeh, Yunzhi Jiang, Yee-Fen Chen, Zhe Chen

**Affiliations:** 1 Integration and Collaboration Laboratory, Department of Industrial Engineering and Engineering Management, National Tsing Hua University, Hsinchu City, Taiwan; 2 School of Automation, Guangdong Polytechnic Normal University, Guangdong, China; 3 Department of Industrial Engineering and Management, Hsiuping University of Science and Technology, Taichung City, Taiwan; 4 School of Information Engineeering, Chang’an University, Xi’an, China; Southwest University, CHINA

## Abstract

The K-harmonic means clustering algorithm (KHM) is a new clustering method used to group data such that the sum of the harmonic averages of the distances between each entity and all cluster centroids is minimized. Because it is less sensitive to initialization than K-means (KM), many researchers have recently been attracted to studying KHM. In this study, the proposed iSSO-KHM is based on an improved simplified swarm optimization (iSSO) and integrates a variable neighborhood search (VNS) for KHM clustering. As evidence of the utility of the proposed iSSO-KHM, we present extensive computational results on eight benchmark problems. From the computational results, the comparison appears to support the superiority of the proposed iSSO-KHM over previously developed algorithms for all experiments in the literature.

## 1. Introduction

Clustering is perhaps the most well-known technique in data mining to cluster data based on certain criteria. In past decades, clustering has attracted much attention, and it is increasingly becoming an important tool due to its wide and valuable applications in improving data analysis in various fields, such as the natural sciences, psychology, medicine, engineering, economics, marketing and other fields [1–28].

Clustering is an NP-hard problem with computational effort growing exponentially with the problem size [1–3]. There are two categories among all existing clustering algorithms: hierarchical clustering and partition clustering [3]. The former builds a hierarchy tree of data that successively merges similar clusters, while the latter begins with a random partition and refines it iteratively [3].

The most popular class of partition clustering is the centroid-based clustering algorithm. Among all clustering methods, with an extensive history dating back to 1972, K-means (KM) is one of the most well-known center-based partition clustering techniques [4–17]. KM is implemented by first randomly selecting *K* initial centroids and then trying to minimize heuristically the sum of the squares of distances, e.g., the Euclidean distance, Manhattan distance, and Mahalanobis distance, between each data point to the centroids [4–16].

As seen above, KM is relatively simple, even on large datasets. Hence, it has effective widespread applications in various real-life problems, such as market segmentation, classification analysis, artificial intelligence, machine learning, image processing, and machine vision [4–16]. Moreover, KM is implemented frequently as a preprocessing stage for other methodologies as a starting configuration.

However, KM is a heuristic algorithm and has two serious drawbacks [7–16]:

1. Its result depends on the initial random clusters, i.e., sensitivity to initial starting centroids;
2. It may be trapped in a local optimum; i.e., there is no guarantee that it will converge to the global optimum.

Therefore, the K-harmonic means (KHM) algorithm was proposed by Zhang [7] in 1999 to solve the problem of sensitivity to initial starting points. However, it still may be trapped by convergence to a local optimum. Hence, the main focus of KHM research has shifted to develop soft computing, such as the tabu K-harmonic means [9], simulated annealing based KHM [10], the particle swarm optimization (PSO) KHM (PSO-KHM) [11], the hybrid data clustering algorithms based on ant colony optimization and KHM [12], a variable neighborhood search (VNS) for KHM clustering [10], the multi-start local search for KHM clustering (MLS) [13], the gravitational search algorithm based KHM [14], the candidate groups search combined with K-harmonic mean (CGS-KHM) [15], the simplified swarm optimization based KHM (SSO-KHM) [16], the statistical feature extraction modeling KHM [29], the PSO hybrid with tabu search for KHM clustering [30], the firefly [31] and the enhanced firefly algorithm [32] for KHM clustering, the fish school search algorithm [33], and the genetic hybrid with gravitational search for KHM clustering [34], to avoid the local trap problem and reduce numerical difficulties.

Soft computing is able to help the traditional KHM methods escape from the local optimum trap and obtain better results [7–16]. However, the update mechanisms of these soft computing methods are either too tedious, which then requires extra computational efforts, or too weak in their local search, which requires more time for convergence [16]. Thus, there is always a need to have a better soft computing method for KHM clustering.

In this paper, a new algorithm, iSSO-KHM, is proposed to help the KHM escape from local optima by installing a new update mechanism into the SSO and integrating the KHM. The rest of the paper is organized as follows: Section 2 provides a description of the KHM and an overview of SSO. The novel one-variable difference update mechanism and the survival of the fittest policy, which are two cores in the proposed iSSO-KHM, are introduced in Section 3. Section 4 compares the proposed iSSO-KHM with three recently introduced KHM-based algorithms in eight benchmark datasets adopted from the UCI database to demonstrate the performance of the proposed iSSO-KHM. Finally, concluding remarks are summarized in Section 5.

## 2. Overview of SSO and KHM

The proposed iSSO-KHM is based on both SSO and KHM. Before discussing the proposed iSSO-KHM, how to solve the KHM clustering, basic SSO and KHM algorithms is introduced formally in this section.

### 2.1 The SSO

SSO is a new population-based soft computing method that was introduced originally by Yeh for discrete-type optimization problems [17] and has applications in two hot research topics in soft computing: swarm intelligence and evolutionary computing. From the applications in various optimization problems, SSO has demonstrated its simplicity, efficiency, and flexibility at exploring large and complex spaces [16–28].

Let Nsol be the number of solutions that are initialized randomly, K be the number of variables and the number of centroids, **c**_*i*_ = (*c*_*i*,1_, *c*_*i*,2_,…, *c*_*i*,*K*_) be the *i*th solution inside the problem space with a fitness value *F*(**c**_*i*_) determined by the fitness function *F* to be optimized, *pBest P*_*i*_ = (*p*_*i*,1_, *p*_*i*,2_,…,*p*_*i*,*K*_) be the best fitness function value of the *i*th solution with its own history, and *gBest P*_*gBest*_ = (*p*_*gBest*,1_, *p*_*gBest*,2_,…,*p*_*gBest*,*K*_) be the solution with the best fitness function value among all *pBest*s, where *i* = 1, 2, …, Nsol and *gBest*∈{1, 2, …, Nsol}.

Analogous to all other soft computing techniques, SSO searches for optimal solutions by updating generations. In every generation of SSO, each variable *c*_*j*,*k*_ is updated according to the following simple step function after *C*_*w*_, *C*_*p*_, and *C*_*g*_ are given:

$$c_{i,k}=\{\begin{array}{l} c_{j,k}\\ p_{i,k}\\ p_{gBest,k}\\ x \end{array}\;\begin{array}{l} {\text{if}\;\rho }_{C}\in [0,C_{w})\\ {\text{if}\;\rho }_{C}\in [C_{w},C_{p})\\ {\text{if}\;\rho }_{C}\in [C_{p},C_{g})\\ {\text{if}\;\rho }_{C}\in [C_{g},\;1) \end{array}.$$ (1)

where *j* = 1, 2, …, Nsol; *k* = 1, 2, …, K; *C*_*w*_, *C*_*p*_*−C*_*w*_, *C*_*g*_, and 1*−C*_*g*_ are the predefined probabilities to determine whether *c*_*j*,*k*_ will be updated to the same value (i.e., no change); *p*_*j*,*k*_ in its *pBest*, *p*_*gBest*,*k*_ of *gBest*, and regenerated to a new randomly generated feasible value [16–28].

Moving toward *pBest* is a local search; moving toward *gBest* is a global search. Moving toward a randomly generated feasible value is also a global search to maintain population diversity and enhance the capacity of escaping from a local optimum. Thus, each solution is a compromise among the current solution, *pBest*, *gBest*, and a random movement; this process combines local search and global search, yielding high search efficiency [16–28].

### 2.2 The KHM

KHM is similar to KM [7–16]. It is also a center-based partition clustering and randomly selects *K* initial centroids in the beginning. The major difference between KHM and KM is that KHM uses harmonic averages of the distances from each data point to the centers as components of its performance function. The detail of the KHM clustering algorithm is shown as follows [7–16]:

#### KHM PROCEDURE

**STEP K1.** Select *K* initial centroids **c**_1_, **c**_2_, …, **c**_*K*_ randomly, where **c**_*k*_ is the centroid of the *k*th cluster; let F^*^ be a large number, and provide a tolerance ε.
**STEP K2.** Calculate fitness function:
  $$F(c_{1},c_{2},\ldots c_{k})=\sum_{i=1}^{N}\frac{K}{\sum_{k=1}^{K}\frac{1}{{\left\|X_{i}-c_{k}\right\|}^{p}}},$$ (2)
  where *p* is the *p*^th^ power of the Manhattan distance.
**STEP K3.** If (F^*^/*F*(**c**_1_, **c**_2_, …, **c**_*K*_) − 1<ε), then halt and go to STEP K7; else, let F^*^ = *F*(**c**_1_, **c**_2_, …, **c**_*K*_).
**STEP K4.** Calculate the membership of each data *X*_*i*_ to centroids **c**_*k*_ for *i* = 1, 2, …, *N* and *k* = 1, 2, …, *K* as below:
  $$M(c_{k},X_{i})=\frac{{\left\|X_{i}-c_{k}\right\|}^{-p-2}}{\sum_{k=1}^{K}{\left\|X_{i}-c_{k}\right\|}^{-p-2}},$$ (3)
**STEP K5.** Calculate the weight of each data *X*_*i*_ for *i* = 1, 2, …, *N* as below:
  $$W(X_{i})=\frac{\sum_{k=1}^{K}{\left\|X_{i}-c_{k}\right\|}^{-p-2}}{(\sum_{k=1}^{K}{\left\|X_{i}-c_{j}\right\|}^{-p}{)}^{2}}.$$ (4)
**STEP K6.** Calculate the new centroid **c**_*k*_ for *k* = 1, 2, …, *K* as below and go to STEP K2:
  $$c_{k}=\frac{\sum_{i=1}^{N}M(c_{k},\;X_{i})\cdot W(X_{i})\cdot X_{i}}{\sum_{i=1}^{N}M(c_{k},\;X_{i})\cdot W(X_{i})}$$ (5)
**STEP K7.** Assign data point *X*_*i*_ to cluster *k* if M(**c**_*j*_, *X*_*i*_)≤M(**c**_*k*_, *X*_*i*_) for *j* = 1, 2, …, *K*.

STEP K2 calculates the fitness function *F*(**c**_1_, **c**_2_, …, **c**_*K*_) of KHM by summing up all harmonic averages of the distances between each data point and all centroids. STEP K3 defines the stopping criteria for KHM. In STEP K4, KHM employs each member function *M*(**c**_*k*_, *X*_*i*_) to measure the influence over the centroid **c**_*k*_ to data *X*_*i*_. This member function determines which cluster each data point belongs to in STEP K7. STEP K5 assigns dynamic weight *W*(*X*_*i*_) to each data point such that the larger the weight is, the smaller the distance is to any centroid to avoid multiple centroids close together. STEP K6 updates the current centroids.

## 3. The Proposed iSSO-KHM

Based on the novel one-variable difference update mechanism and the policy of survival of the fittest, the proposed iSSO-KHM is able to find a good solution without needing to explore all possible combinations of solutions. These two parts, i.e., the novel one-variable difference update mechanism and the policy of survival of the fittest, are discussed in this section.

### 3.1 The one-variable difference update mechanism

Each soft computing method has its own generic update mechanism and numerous revised update mechanisms for different applications in various situations. In most soft computing, the update mechanism is only changed slightly. For example, the update mechanism of PSO is considered to be a vector-based update mechanism using the following two equations where *c*_1_ and *c*_2_ are two constants:

$$V_{i}^{t+1}=wV_{i}^{t}+c_{1}\cdot \rho_{1}\cdot (P_{i}^{t}-X_{i}^{t})+c_{2}\cdot \rho_{2}\cdot (P_{gBest}^{t}-X_{i}^{t})$$ (6)

$$X_{i}^{t+1}=X_{i}^{t}+V_{i}^{t+1}.$$ (7)

Note that all variables in the same solution share two random variables in PSO, i.e., ρ_1_ and ρ_2_ which are generated randomly from a uniform distribution within [0, 1] in Eq 6. In ABC, one variable for each solution is selected randomly for updating. The updated operators in traditional GA are either two variables via one-cut-point mutation, or up to half the number of variables changed via one-cut-point crossover. In the traditional SSO, however, all variables are updated simultaneously based on Eq 1.

To reduce the number of random values and to change solutions gradually without breaking the trend and stability in the convergent status, only one variable is updated in each solution for each iteration in the proposed iSSO-KHM. Another reason to adapted the one-variable update mechanism is due to the specific factor that the KHM is essentially insensitive to the initial conditions and only needs to refine its solution [7–16].

The update mechanism listed in Eq 1 is more suitable for this discrete data or type, and each variable of centroids is a floating point value in the KHM. Hence, the step function in Eq 1 is also revised for floating-point data in the novel one-variable difference update mechanism for the proposed iSSO-KHM as follows:

$$c_{j}{}_{,k}=c_{j}{}_{,k}+\rho_{1}-\rho_{2}\cdot \{\begin{array}{ll} \left(c_{gBest,k}-c_{j,k}\right) & {\text{if}\;\rho }_{C}\in [0,C_{g})\\ \left(c_{gBest,k}-c_{y,k}\right) & {\text{if}\;\rho }_{C}\in [C_{g},C_{w})\\ \left(c_{x,k}-c_{j,k}\right) & \text{otherwise} \end{array},$$ (8)

where ρ_1_, ρ_2_, and ρ_c_ are random numbers generated from the uniform distribution within [0,1]. Note that *C*_*g*_ = .4 and *C*_*w*_ = .6 in this study, the role of *pBest* is removed, and the comparison order is *C*_*g*_ first and then *C*_*w*_ in the step function of Eq 6, which is different from Eq 1.

For example, let **c**_3_ = (1.3, 4.5, 6.7, 8.9) be the current solution, **c**_*gBest*_ = **c**_6_ = (2.7, 7.6, 5.4, 9.8) be the *gBest*, **c**_*x*_ = **c**_5_ = (2.3, 5.5, 7.7, 9.9) and **c**_*y*_ = **c**_7_ = (6.2, 8.5, 1.7, 4.9) be two randomly selected solutions, and the third variable (i.e., *c*_3,3_) be selected randomly to update. Assume that ρ_1_ = 0.3 and ρ_2_ = 0.6 are generated randomly. Table 1 shows the newly updated **c**_3_ for three different cases resulting from three different values of ρ_c_:

**Table 1:** The new update c_3_ for three different cases.

Case	ρ_c_	original *c*_3,3_	updated *c*_3,3_	updated c_3_
1	0.12	6.7	6.7+0.3∙0.6∙(5.4−6.7) = 6.466	(1.3, 4.5, **6.466**, 8.9)
2	0.45	6.7	6.7+0.3∙0.6∙(5.4−1.7) = 7.366	(1.3, 4.5, **7.366**, 8.9)
3	0.99	6.7	6.7+0.3∙0.6∙(7.7−1.7) = 7.780	(1.3, 4.5, **7.780**, 8.9)

### 3.2 Survival-of-the-fittest policy

The policy of survival of the fittest, inspired by natural selection, is a strategy to select the most fits and eliminate unfits. In the traditional SSO, the updated solution must replace the old solution regardless of whether the updated solution is worse [17–28]. However, *gBest* is based on survival-of-the-fittest policy; i.e., only a solution that is better than the *gBest* can replace *gBest* [17–28].

Unlike SSO, the proposed one-variable difference update mechanism only updates one variable and places more emphasis on the local search. Additionally, KHM is less sensitive to the updated solutions. Hence, the survival-of-the-fittest policy applies to both *gBest* and all updated solutions to reduce the evolution time.

### 3.3 The complete pseudocode of the proposed iSSO-KHM

Like the existing related KHM algorithms, the KHM procedure discussed in section 2.2 to calculate the fitness of each solution is implemented in the iSSO-KHM and acts as a local search to further improve each updated solution heuristically. The steps of complete pseudocode of the proposed iSSO-KHM are described as follows.

#### iSSO-KHM PROCEDURE

**STEP 0.** Generate **c**_*j*_ = (*c*_*j*,1_, *c*_*j*,2_, …, *c*_*j*,*K*_) randomly, update **c**_*j*_, and calculate its fitness using the KHM procedure discussed in Section 2.2 for all *j* = 1, 2, …, Nsol.
**STEP 1.** Let gen = 1 and find *gBest*∈{1, 2, …, Nsol} such that *F*(**c**_*gBest*_)≤*F*(**c**_*j*_) for all *j* = 1, 2, …, Nsol.
**STEP 2.** Let *j* = 1.
**STEP 3.** Select a variable (i.e., a centroid) randomly from **c**_*j*_, say *c*_*j*,*k*_ where *k*∈{1, 2, …, K}, and let c^*^ = **c**_*j*_ and F^*^ = *F*(**c**_*j*_).
**STEP 4.** Generate a random number ρ_*C*_ from the uniform distribution between [0, 1]
**STEP 5.** If ρ_*C*_<*C*_*g*_, then let *x* = *gBest*, *y* = *j*, and go to STEP 8.
**STEP 6.** If ρ_*C*_<*C*_*w*_, then let *x* = *gBest*, select *y* randomly from {1, 2, …, *K*}, and go to STEP 8.
**STEP 7.** Select two integers *x* and *y* randomly from {1, 2, …, *K*}.
**STEP 8.** Let *c*_*j*,*k*_ = *c*_*j*,*k*_+ρ_[0,1]_∙ρ_[0,1]_+(*c*_*x*,*k*_−*c*_*y*,*k*_), and run the procedure KHM to update **c**_*j*_ and calculate its fitness.
**STEP 9.** If *F*(**c**_*j*_)>F^*^, then let **c**_*j*_ = c^*^ and *F*(**c**_*j*_) = F^*^, and go to STEP 11.
**STEP 10.** If *F*(**c**_*j*_)<*F*(**c**_*gBest*_), then let *gBest* = *j*.
**STEP 11.** If the runtime is less than the predefined *T*, then go to STEP 2; otherwise, **c**_*gBest*_ is the final solution, and halt.
**STEP 12.** If *j*<Nsol, let *j* = *j*+1, and go to STEP 3.

In the above, STEP 0 simply runs the KHM procedure for each randomly generated solution to calculate its fitness function and update the solution. STEP 1 finds the first *gBest* from these initial populations after using the KHM procedure. STEPs 2–12 implement the proposed one-variable difference update mechanism; STEPs 9 and 10 are based on the survival-of-the-fittest policy to decide whether to accept the updated solution or replace *gBest*. Note that the stopping criterion in STEP 11 is the runtime T, and T = 0.1, 0.3, and 0.5 CPU seconds in the experiments tested in Section 4.

## 4. Experimental Results

In this section, we present the computational results of the comparisons among the proposed algorithm and existing algorithms on eight benchmark datasets to test the performance of iSSO-KHM.

### 4.1 The Experimental Setting

To evaluate the efficiency and effectiveness (i.e., the solution quality) of the proposed iSSO-KHM, eight benchmarks adopted from UCI are tested: Abalone (denoted by A, 4177 records and seven features), Breast-Cancer-Wisconsin (denoted by B, 699 records and nine features), Car (denoted by C, 1728 records and six features), Glass (denoted by G, 214 records and nine features), Iris (denoted by I, 150 records and four features), Segmentation (denoted by S, 2310 records and 19 features), Wine (denoted by W, 178 records and 13 features), and Yeast (denoted by Y, 1484 records and eight features).

Moreover, iSSO-KHM is compared to four KHM-related soft computing algorithms: CGS_KHM, MLS_KHM, PSO_KHM, and SSO_KHM. Note that CGS_KHM has better performance than tabu search and VNS for the Iris, Glass and Wine datasets.

The programming language used was C++ with default options for all five algorithms: CGS_KHM (denoted by CGS), iSSO-KHM (denoted by iSSO), MLS_KHM (denoted by MLS), PSO_KHM (denoted by MLS), and SSO_KHM (denoted by SSO). All codes were run using a 64-bit Window 10 Operating System with Intel Core i7-5960X 3.00 GHz CPU and 16 GB of RAM.

In experiments, all values of K are set to three; the *p*^th^ power of the Manhattan distance is *p* = 1.5, 2.0, and 3.0; and the runtime limit is *T* = 0.1, 0.3, and 0.5 CPU seconds. For each test and algorithm, the number of solutions is 15, i.e., Nsol = 15, the number of independent runs is 55, and only the best 50 results are recorded to remove possible outliers; the stopping criteria are *T* = 0.1, 0.3, and 0.5 CPU seconds.

All required parameters for CGS, MLS, PSO, and SSO are taken directly from [15], [13], [11], and [20] for a fair comparison; two parameters, *C*_*g*_ = 0.4 and *C*_*w*_ = 0.6, are used in the proposed iSSO-KHM.

In all tables listed in S1 Appendix and the following two subsections, the notations F_avg_, F_min_, F_max_, and F_std_ denote the average, minimal (the best), maximal (the worst) and standard deviation of the fitness values obtained from related algorithms. Additionally, the notations f_avg_, f_min_, f_max_ and f_std_ represent the number of F_avg_, F_min_, F_max_ and F_std_ that are the best among all algorithms under the same related conditions, e.g., *p*, *T*, and/or dataset.

To compare the efficiency of the update mechanism of the proposed iSSO, the average of the corresponding fitness calculation number (N_avg_) and the number of best N_avg_ represented by n_avg_ are recorded. Note that for a fixed *T*, a higher N_avg_ means that the related update mechanism is more efficient and increases the search performance for finding an optimal solution.

To properly evaluate the clustering method, the F_measure_ value is provided and the number of best F_measure_ [35,36] is represented by f_mea_. The F_measure_ is one of the standard clustering validity measures based on the ideas of precision and recall from information retrieval [35,36]. Evidently, the bigger value of F_measure_ is, the higher the quality of clustering is.

All experimental results are listed in S1 Appendix. S1 Appendix demonstrates that iSSO has achieved better solutions for each test problem with lower standard deviations and higher fitness computation numbers compared to the other methods.

### 4.2 General Observations for f_avg_, f_min_, f_max_, f_std_, and n_avg_, and f_mea_

All results in S1 Appendix are ranked and discussed in this subsection. Tables 2–5 summarize these ranking based on different *T* and *p*, *T* only, *p* only, and algorithm only, respectively. The letter next to the number denotes the related dataset, e.g., B2S denotes one best value in dataset B and two best value in dataset S.

**Table 2:** The number of f_avg_, f_min_, f_max_, f_std_, n_avg_, and f_mea_.

	*T*			0.1						0.3						0.5			
*p*	Alg.	f_avg_	f_min_	f_max_	f_std_	n_avg_	f_mea_	f_avg_	f_min_	f_max_	f_std_	n_avg_	f_mea_	f_avg_	f_min_	f_max_	f_std_	n_avg_	f_mea_
1.5	CGS	S	0	S	BS	0	0	S	0	S	S	0	W	0	0	0	0	0	0
	iSSO	**6**	**6**	**7**	**6**	**7**	**5**	**7**	**7**	**7**	**7**	**8**	**7**	**8**	**8**	**8**	**8**	**8**	**7**
	MLS	0	S	0	0	A	0	0	0	0	0	0	0	0	0	0	0	0	S
	PSO	0	0	0	0	0	W	0	0	0	0	0	0	0	0	0	0	0	0
	SSO	A	A	0	0	0	AS	0	A	0	0	0	0	0	0	0	0	0	0
2.0	CGS	S	S	S	BS	0	0	S	0	S	S	0	0	0	0	0	0	0	0
	iSSO	**7**	**6**	**7**	**6**	**7**	**6**	**6**	**8**	**6**	**6**	**8**	**5**	**7**	**8**	**7**	**7**	**8**	**5**
	MLS	0	0	0	0	A	W	B	0	B	B	0	W	B	0	B	B	0	0
	PSO	0	0	0	0	0	0	0	0	0	0	0	S	0	0	0	0	0	I
	SSO	0	A	0	0	0	A	0	0	0	0	0	A	0	0	0	0	0	AY
2.5	CGS	S	0	S	BS	0	0	0	0	S	S	0	0	0	0	0	0	0	0
	iSSO	**7**	**6**	**7**	**6**	**7**	**5**	**8**	**8**	**7**	**7**	**8**	**5**	**8**	**8**	**8**	**8**	**8**	**6**
	MLS	0	S	0	0	A	W	0	0	0	0	0	0	0	0	0	0	0	G
	PSO	0	0	0	0	0	IS	0	0	0	0	0	BW	0	0	0	0	0	0
	SSO	0	A	0	0	0	0	0	0	0	0	0	A	0	0	0	0	0	I

**Table 3:** The values of f_avg_, f_min_, f_max_, f_std_, n_avg_, and f_mea_ for *T* = 0.1, 0.3, and 0.5.

*T*	Alg.	f_avg_	f_min_	f_max_	f_std_	n_avg_	f_mea_
	CGS	3 (S)	1 (S)	3 (S)	6 (3B,3S)	0	0
	iSSO	**20**	**18**	**21**	**18**	**21**	**16**
0.1	MLS	0	2 (S)	0	0	3 (A)	2 (W)
	PSO	0	0	0	0	0	3 (I,S,W)
	SSO	1 (A)	3 (A)	0	0	0	2 (A,S)
	CGS	2 (S)	0	3 (S)	3 (S)	0	1 (W)
	iSSO	**21**	**23**	**20**	**20**	**24**	**17**
0.3	MLS	1 (B)	0	1 (B)	1 (B)	0	1 (W)
	PSO	0	0	0	0	0	3 (B,S,W)
	SSO	0	1 (A)	0	0	0	2 (A)
	CGS	0	0	0	0	0	0
	iSSO	**23**	**24**	**23**	**23**	**24**	**18**
0.5	MLS	1 (B)	0	1 (B)	1 (B)	0	2 (G,S)
	PSO	0	0	0	0	0	1 (I)
	SSO	0	0	0	0	0	3 (A,I,Y)

**Table 4:** The values of f_avg_, f_min_, f_max_, f_std_, n_avg_, and f_mea_ for *p* = 1.5, 2.0, and 2.5.

*p*	Alg.	f_avg_	f_min_	f_max_	f_std_	n_avg_	f_mea_
1.5	CGS	2 (S)	0	2 (S)	3 (B,2S)	0	1 (W)
	iSSO	**21**	**21**	**22**	**21**	**23**	**19**
	MLS	0	1 (S)	0	0	1 (A)	1 (S)
	PSO	0	0	0	0	0	1 (W)
	SSO	1 (A)	2 (A)	0	0	0	2 (A,S)
2	CGS	2 (S)	1 (S)	2 (S)	3 (B,2S)	0	0
	iSSO	**20**	**22**	**20**	**19**	**23**	**16**
	MLS	2 (B)	0	2 (B)	2 (B)	1 (A)	2 (W)
	PSO	0	0	0	0	0	2 (I,S)
	SSO	0	1 (A)	0	0	0	4 (3A,Y)
2.5	CGS	1 (S)	0	2 (S)	3 (B,2S)	0	0
	iSSO	**23**	**22**	**22**	**21**	**23**	**16**
	MLS	0	1 (S)	0	0	1 (A)	2 (G,W)
	PSO	0	0	0	0	0	4 (B,I,S,W)
	SSO	0	1 (A)	0	0	0	2 (A,I)

**Table 5:** The values of f_avg_, f_min_, f_max_, f_std_, n_avg_, and f_mea_ for algorithms.

Alg.	f_avg_	f_min_	f_max_	f_std_	n_avg_	f_mea_
CGS	5 (S)	0	6 (S)	9 (3B,6S)	0	1 (W)
iSSO	**64**	**65**	**64**	**61**	**69**	**51**
MLS	2 (B)	2 (S)	2 (B)	2 (B)	3 (A)	5 (G, 3W, S)
PSO	0	0	0	0	0	7 (B, 2I, 2S, 2W)
SSO	1 (A)	4 (A)	0	0	0	8 (5A, I, S, Y)

From Table 2, iSSO has higher numbers in f_avg_, f_min_, f_max_, f_std_, n_avg_, and f_mea_ than other methods for different setting of *T* and *p*. Hence, iSSO is more efficient, effective, and robust than other methods.

Table 3 summarizes the values of f_avg_, f_min_, f_max_, f_std_, n_avg_, and f_mea_ for *T* = 0.1, 0.3, and 0.5 separately. We can observe that the longer runtime is, the better the solution quality obtained from iSSO in Table 3. For example, f_min_ is increased from 18 to 23 for *T* = 0.1 to *T* = 0.2. PSO is the second best in f_mea_ for both *T* = 0.1 and 0.3; SSO is the second best in f_min_ for T = 0.1 and 0.2, and in f_mea_ for *T* = 0.3. Additionally, as seen from Table 3, iSSO tends to perform much better than other methods from time to time, e.g., there are six cases in which F_min_ are better than that of iSSO for *T* = 0.1 but none in which F_min_ is better than that of iSSO for *T* = 0.3.

Table 4 sums up the values of f_avg_, f_min_, f_max_, f_std_, n_avg_, and f_mea_ for *p* = 1.5, 2.0, and 2.5 separately. It is evident that iSSO is still the best method compared to the others in all aspects. According to published results, other methods work more effectively when p = 2.0 [7–16]. However, given the results, iSSO still retains its performance, regardless of the value of *p*. For example, f_min_ is 21 for *p* = 1.5 and 22 for both *p* = 2.0 and 2.5. Interesting observations can still be found, as observed in Table 3, where, in general, CGS yields better results for the S dataset, than other datasets. PSO and SSO follow on in performance with f_mea_ in *p* = 2.0 and *p* = 2.5, respectively.

Table 5 lists the overall values of f_avg_, f_min_, f_max_, f_std_, n_avg_, and f_mea_ for CGS, iSSO, MLS, PSO, and SSO separately. In general, it seems that iSSO is only slightly more powerful within dataset A as f_min_ = 4 and f_mea_ = 5 for SSO and for within dataset S as f_min_ = 2 for MLS, f_mea_ = 2 for PSO, and f_avg_ = 5 and f_max_ = f_std_ = 6 for CGS. This is similar to what is observed in Tables 2 and 3. However, the number of best values for iSSO in all statistical indexes are still more than 6.2 times better compared to those of other methods. For example, for f_std_, CGS produced nine best values (3 in B dataset and 6 in S dataset), whereas iSSO produced 64 best values. This trend is also found when iSSO is compared across all algorithms and thus demonstrates that iSSO outperforms the other algorithms in almost all aspects.

### 4.3 General Observations for F_avg_, F_min_, F_max_, F_std_, N_avg_, and F_measure_

In general, each result obtained using the proposed iSSO is better than those obtained using the other methods described S1 Appendix and Section 4.2. For an elaborate analysis, the top five values of F_min_ for each dataset under all settings of *T* and *p* are summarized in Table 6 and discussed in this subsection.

**Table 6:** The top five F_min_ for each dataset.

ID	*T*	*p*	Alg.	F_avg_	F_min_	F_max_	F_std_	N_avg_	F_measure_
A	0.5	2.5	iSSO	377.100	377.096	377.115	3.92E-03	254.26	57.70%
	0.3	2.5	iSSO	377.129	377.097	377.226	3.42E-02	152.28	58.64%
	0.3	2.5	SSO	380.591	377.116	403.228	5.83E+00	146.32	58.67%
	0.5	2.5	SSO	385.472	377.157	414.058	1.04E+01	241.32	57.69%
	0.5	2.5	PSO	418.457	377.634	426.647	1.96E+01	238.2	57.59%
B	0.5	1.5	iSSO	424.867	424.867	424.867	1.12E-06	1685.48	96.15%
	0.3	1.5	iSSO	424.867	424.867	424.867	1.71E-05	1023.24	96.12%
	0.1	2.5	iSSO	424.886	424.867	424.972	2.66E-02	356.56	96.16%
	0.1	2	iSSO	424.883	424.867	424.998	2.42E-02	357.28	96.14%
	0.1	1.5	iSSO	424.888	424.868	424.967	2.71E-02	341.78	96.19%
C	0.5	1.5	iSSO	7068.628	7068.628	7068.628	8.33E-06	722.9	39.28%
	0.3	1.5	iSSO	7068.630	7068.628	7068.632	1.08E-03	436.18	39.25%
	0.1	2.5	iSSO	7069.794	7069.146	7070.432	3.35E-01	150.64	39.33%
	0.1	2	iSSO	7070.008	7069.150	7070.889	4.04E-01	150.66	39.39%
	0.1	1.5	iSSO	7069.977	7069.189	7070.878	4.25E-01	144.18	39.23%
G	0.3	1.5	iSSO	1059.336	1059.336	1059.336	7.59E-09	5099.84	41.42%
	0.3	2	iSSO	1059.336	1059.336	1059.336	6.87E-09	5100.06	41.33%
	0.1	2.5	iSSO	1059.336	1059.336	1059.337	6.21E-06	1780.36	41.39%
	0.1	1.5	iSSO	1059.336	1059.336	1059.337	5.95E-06	1705.48	41.39%
	0.1	2	iSSO	1059.336	1059.336	1059.337	9.23E-06	1779.64	41.50%
I	0.3	1.5	iSSO	181.728	181.728	181.728	2.04E-11	9494.02	75.31%
	0.3	2	iSSO	181.728	181.728	181.728	1.83E-11	9495.64	75.36%
	0.1	1.5	iSSO	181.728	181.728	181.728	1.08E-07	3185.04	75.25%
	0.1	2	iSSO	181.728	181.728	181.728	7.83E-08	3313.24	75.41%
	0.1	2.5	iSSO	181.728	181.728	181.728	6.70E-08	3316.62	75.26%
S	0.5	1.5	iSSO	42852347.416	42852345.652	42852350.697	1.51E+00	412.40	53.18%
	0.5	2	iSSO	42852347.460	42852345.678	42852351.912	1.61E+00	412.80	53.10%
	0.3	2	iSSO	42852454.042	42852348.975	42852724.919	9.39E+01	248.00	53.19%
	0.3	1.5	iSSO	42852441.557	42852356.230	42852682.740	7.27E+01	247.90	53.07%
	0.3	2	CGS	42852405.328	42852368.882	42852456.610	2.33E+01	184.10	53.14%
W	0.3	1.5	iSSO	5388248.279	5388248.279	5388248.279	4.70E-09	5095.64	62.07%
	0.3	2	iSSO	5388248.279	5388248.279	5388248.279	4.70E-09	5095.72	62.22%
	0.1	2	iSSO	5388248.279	5388248.279	5388248.279	4.69E-08	1776.94	62.15%
	0.1	1.5	iSSO	5388248.279	5388248.279	5388248.279	1.21E-07	1704.06	62.20%
	0.1	2.5	iSSO	5388248.279	5388248.279	5388248.279	6.11E-08	1779.74	62.08%
Y	0.5	2.5	iSSO	72.833	72.833	72.836	9.49E-04	769.6	56.21%
	0.3	2.5	iSSO	72.837	72.833	72.857	5.41E-03	465.74	56.15%
	0.3	2.5	SSO	74.094	72.952	75.856	8.53E-01	445.44	56.05%
	0.5	2.5	SSO	73.942	73.038	75.630	6.96E-01	734.42	56.09%
	0.3	2.5	PSO	74.343	73.185	75.418	6.40E-01	399.8	55.84%

In Table 6, the proposed iSSO has the largest number (33) of results among the top five values, and SSO, PSO, and CGS have four, two, and one results among the top five values of F_min_, respectively. Note that in most cases SSO yields better results than CGS and MLS, as seen in Table 6, but all of the values of f_avg_, f_min_, f_max_, f_std_ are zero for SSO in Section 4.2.

Additionally, we can see that the top four F_min_ values are all obtained from the proposed iSSO for all datasets, except iSSO only has the top two F_min_ in both A and S datasets, of which SSO has the 3^rd^ and 4^th^ best F_min_, and PSO has the 5^th^ best F_min_. It seems that the algorithm with the best F_min_ also has the best F_avg_, F_max_, F_std_, and N_avg_ in all datasets. However, the algorithm with the best F_min_ does not guarantee its F_measure_ is also the best, this is applicable to A, B, C, G, I and W datasets.

The following are some other observations for *p*, *T*, N_avg_, and F_std_:

1. *p*: There are 14, 11, and 15 top-five F_min_ values across all the eight data sets for *p* = 1.5, 2.0, and 2.5 in Table 6, respectively. This debunks published literatures [7–16] which indicate that *p* = 2.0 yields the best result. The above observation is also found in Table 4 of Section 4.2.
2. *T*: The *T* = 0.1, 0.3, and 0.5 have 15, 16, and 9 top-five F_min_ values across all the eight data sets. Among top-five F_min_ values for each dataset, the one with the largest *T* also has the best F_min_, e.g., *T* = .3 in G, I and W datasets and *T* = 0.5 in the rest of the datasets. Hence, the above result agrees with the basic concept in soft computing: more runtime results in better solution quality.
3. N_avg_: The order of the best N_avg_ for each dataset from large to small is 9494.02 (I) > 5099.84 (G) > 5095.64 (W) > 1685.48 (B) > 769.6 (Y) > 722.9 (C) > 412.40 (S) > 254.26 (A), where the letter inside parentheses is the related dataset. The above order exactly coincides with the order from large to small of the number of recorders in each dataset: A (4177) > S (2310) > C (1728) > Y (1484) > B (699) > G (214) > W (178) > I (150), except when 5099.84 (G) > 5095.64 (W) in N_avg_. Hence, the smaller the dataset is, the shorter the runtime is and the larger number of fitness calculations is.
4. F_std_: The order of the best F_std_ for each dataset from small to large is 2.04E-11 (I) < 4.70E-09 (W) < 7.59E-09 (G) < 1.12E-06 (B) < 8.33E-06 (C) < 9.49E-04 (Y) < 3.92E-03 (A) < 1.51E+00 (S), where the letter inside parentheses is the related dataset. The above order of datasets is similar to that of N_avg_ because the more fitness calculations are performed, the lower is standard deviation.

## 5. Conclusions

In this work, a new soft computing method called the iSSO-KHM is proposed to solve the KHM clustering problem. The proposed iSSO-KHM adapted the fundamental concepts in both the traditional SSO and KHM by adding the novel one-variable difference update mechanism to update solutions and the survival-of-the-fittest policy to decide whether to accept the new update solutions.

The computational experiments compare the proposed iSSO-KHM with CGS, MLS, PSO, and SSO on eight benchmark datasets: Abalone, Breast-Cancer-Wisconsin, Car, Glass, Iris, Segmentation, Wine, and Yeast with settings of *K* = 3; *p* = 1.5, 2.0, and 2.5; and *T* = 0.1, 0.3, and 0.5.

The experimental results show the superiority of iSSO-KHM over the other three algorithms for almost all eight benchmark datasets. Hence, iSSO-KHM can achieve a trade-off between exploration and exploitation to generate a good approximation in a limited computation time systematically, efficiently, effectively, and robustly.

However, from the experiments in Section 4, the improved F_min_ value does not mean that the F_measure_ is also improved. Therefore, a potential area of exploration would be to include F_measure_ in the fitness function to improve both values of F_min_ and F_measure_. Another limitation of the proposed algorithm is that *C*_*g*_ and *C*_*w*_ in Eq 8 of the proposed update mechanism must be known in advance, this also brings up another practical problem that is to develop a parameter free idea in the proposed algorithm in the future.

As there are some recently proposed swarm-based clustering algorithms, it is necessary to have more comparisons about the proposed algorithm with other well-known swarm-based clustering algorithms in the future. In Section 4, “Experimental results”, the choice of the parameter *K* is fixed to 3. The proposed approach will also compare with the other versions of KHM for different values of *K* (like the case of *p* and *T* parameters).

## Supporting Information

S1 Appendix (DOCX)
