# Correction: A New Soft Computing Method for K-Harmonic Means Clustering

**DOI:** 10.1371/journal.pone.0169707

**Published:** 2017-01-03

**Authors:** Wei-Chang Yeh, Yunzhi Jiang, Yee-Fen Chen, Zhe Chen

There are errors in the Funding section. The correct funding information is as follows: This research was supported in part by the Ministry of Science and Technology, R.O.C. under grant MOST 104-2221-E-007-061-MY3. The funders had no role in study design, data collection and analysis, decision to publish, or preparation of the manuscript.
