# Supplementary material for: A New Soft Computing Method for K-Harmonic Means Clustering
Source: PLoS One. 2016 Nov 15;11(11):e0164754. doi: 10.1371/journal.pone.0164754 (PMC5112810; doi:10.1371/journal.pone.0164754)
Supplement: S1 Appendix — (DOCX) [file pone.0164754.s001.docx]

## S1 Appendix

#### **Table A1.** Experimental results for *T*=.1 and *p*=1.5.

| ID | Alg. | F_avg_ | F_min_ | F_max_ | F_std_ | N_avg_ | F_measure_ |
| --- | --- | --- | --- | --- | --- | --- | --- |
| A | CGS | 1127.631 | 1011.289 | 1237.817 | 5.84E+01 | 30.0 | 56.05% |
|  | iSSO | 979.007 | 953.105 | **1005.300** | **1.30E+01** | 50.8 | 59.48% |
|  | MLS | 1105.484 | 963.912 | 1186.508 | 5.20E+01 | **51.5** | 58.82% |
|  | PSO | 1079.474 | 951.734 | 1082.581 | 5.12E+01 | 51.3 | 59.60% |
|  | SSO | **977.695** | **951.113** | 1015.889 | 1.46E+01 | 51.0 | **59.71%** |
| B | CGS | 425.032 | 425.016 | 425.065 | **1.39E-02** | 122.3 | 96.13% |
|  | iSSO | **424.888** | **424.868** | **424.967** | 2.71E-02 | **341.8** | **96.19%** |
|  | MLS | 425.047 | 425.017 | 425.103 | 2.25E-02 | 306.0 | 96.18% |
|  | PSO | 646.107 | 429.222 | 816.273 | 1.46E+02 | 320.9 | 95.21% |
|  | SSO | 683.584 | 430.157 | 920.421 | 1.71E+02 | 335.9 | 94.99% |
| C | CGS | 7104.376 | 7090.115 | 7113.286 | 6.01E+00 | 41.9 | 39.06% |
|  | iSSO | **7069.977** | **7069.189** | **7070.878** | **4.25E-01** | **144.2** | **39.23%** |
|  | MLS | 7101.181 | 7083.257 | 7110.712 | 5.88E+00 | 133.6 | 39.19% |
|  | PSO | 7101.831 | 7082.524 | 7145.373 | 1.07E+01 | 139.4 | 39.15% |
|  | SSO | 7118.262 | 7076.745 | 7152.248 | 2.40E+01 | 140.4 | 39.20% |
| G | CGS | 1084.481 | 1065.105 | 1104.281 | 1.30E+01 | 72.9 | 41.11% |
|  | iSSO | **1059.336** | **1059.336** | **1059.337** | **5.95E-06** | **1705.5** | **41.39%** |
|  | MLS | 1066.186 | 1062.227 | 1072.286 | 1.97E+00 | 1406.7 | 41.25% |
|  | PSO | 1114.237 | 1062.107 | 1103.309 | 2.99E+01 | 1413.5 | 41.38% |
|  | SSO | 1134.441 | 1060.784 | 1243.670 | 4.29E+01 | 1595.5 | 41.37% |
| I | CGS | 184.697 | 181.883 | 188.405 | 2.02E+00 | 53.4 | 75.09% |
|  | iSSO | **181.728** | **181.728** | **181.728** | **1.08E-07** | **3185.0** | **75.25%** |
|  | MLS | 182.123 | 181.848 | 182.546 | 1.91E-01 | 2826.1 | 75.20% |
|  | PSO | 191.301 | 181.755 | 229.042 | 2.38E+00 | 2949.1 | 75.25% |
|  | SSO | 194.996 | 181.746 | 285.865 | 2.06E+01 | 2971.3 | 75.22% |
| S | CGS | **42852460.136** | 42852373.736 | **42852587.786** | **6.09E+01** | 72.1 | 53.05% |
|  | iSSO | 42914593.398 | 42854211.562 | 43125746.672 | 6.30E+04 | **79.9** | 53.12% |
|  | MLS | 42852562.195 | **42852372.399** | 42852827.596 | 1.11E+02 | 72.2 | 53.07% |
|  | PSO | 44355831.290 | 42857959.654 | 44999596.055 | 1.69E+06 | 73.8 | 53.04% |
|  | SSO | 47589151.997 | 42906459.465 | 51367457.800 | 3.14E+06 | 77.3 | **53.14%** |
| W | CGS | 5388292.312 | 5388254.633 | 5388305.710 | 1.67E+01 | 348.3 | 62.14% |
|  | iSSO | **5388248.279** | **5388248.279** | **5388248.279** | **1.21E-07** | **1704.1** | 62.20% |
|  | MLS | 5388286.003 | 5388251.780 | 5388303.866 | 1.62E+01 | 1385.3 | 62.15% |
|  | PSO | 5557214.495 | 5388847.463 | 6039842.851 | 4.90E+04 | 1511.6 | **62.22%** |
|  | SSO | 5575017.601 | 5393542.194 | 6169083.778 | 1.86E+05 | 1533.5 | 62.21% |
| Y | CGS | 123.027 | 122.352 | 123.289 | 1.96E-01 | 30.0 | 55.09% |
|  | iSSO | **121.677** | **121.664** | **121.692** | **7.57E-03** | **214.2** | **55.54%** |
|  | MLS | 122.766 | 122.278 | 123.018 | 1.78E-01 | 172.4 | 55.18% |
|  | PSO | 122.716 | 121.957 | 123.444 | 2.75E-01 | 189.1 | 55.43% |
|  | SSO | 122.509 | 121.788 | 123.783 | 5.55E-01 | 203.3 | 55.39% |

#### **Table A2.** Experimental results for *T*=.1 and *p*=2.0.

| ID | Alg. | F_avg_ | F_min_ | F_max_ | F_std_ | N_avg_ | F_measure_ |
| --- | --- | --- | --- | --- | --- | --- | --- |
| A | CGS | 1123.423 | 995.881 | 1231.387 | 5.25E+01 | 30.0 | 56.03% |
|  | iSSO | **975.318** | 950.668 | **1001.924** | **1.15E+01** | 51.7 | 58.81% |
|  | MLS | 1123.883 | 1009.794 | 1209.724 | 5.03E+01 | **52.6** | 55.35% |
|  | PSO | 1083.446 | 958.645 | 1607.711 | 2.06E+02 | 52.5 | 58.30% |
|  | SSO | 1026.798 | **950.545** | 2051.054 | 2.07E+02 | 52.1 | **58.85%** |
| B | CGS | 425.035 | 425.017 | 425.069 | **1.50E-02** | 127.3 | 96.10% |
|  | iSSO | **424.883** | **424.867** | **424.998** | 2.42E-02 | **357.3** | **96.14%** |
|  | MLS | 425.049 | 425.017 | 425.106 | 2.78E-02 | 317.6 | 96.11% |
|  | PSO | 507.105 | 428.052 | 578.165 | 1.42E+02 | 321.0 | 95.52% |
|  | SSO | 680.211 | 428.542 | 920.421 | 1.85E+02 | 349.6 | 95.44% |
| C | CGS | 7104.657 | 7087.793 | 7113.486 | 5.56E+00 | 42.0 | 39.10% |
|  | iSSO | **7070.008** | **7069.150** | **7070.889** | **4.04E-01** | **150.7** | **39.39%** |
|  | MLS | 7101.458 | 7088.206 | 7111.551 | 4.82E+00 | 133.5 | 39.10% |
|  | PSO | 7116.895 | 7083.115 | 7132.608 | 2.64E+01 | 143.2 | 39.17% |
|  | SSO | 7124.205 | 7082.772 | 7163.005 | 2.68E+01 | 146.0 | 39.25% |
| G | CGS | 1080.735 | 1066.070 | 1102.491 | 1.10E+01 | 73.6 | 41.13% |
|  | iSSO | **1059.336** | **1059.336** | **1059.337** | **9.23E-06** | **1779.6** | **41.50%** |
|  | MLS | 1066.052 | 1061.469 | 1070.441 | 1.97E+00 | 1463.7 | 41.38% |
|  | PSO | 1095.491 | 1061.693 | 1140.402 | 9.07E+00 | 1664.8 | 41.30% |
|  | SSO | 1138.486 | 1062.995 | 1275.125 | 4.51E+01 | 1672.0 | 41.27% |
| I | CGS | 185.597 | 182.017 | 189.461 | 2.18E+00 | 53.3 | 75.15% |
|  | iSSO | **181.728** | **181.728** | **181.728** | **7.83E-08** | **3313.2** | **75.41%** |
|  | MLS | 182.143 | 181.792 | 182.707 | 2.20E-01 | 2946.0 | 75.34% |
|  | PSO | 188.885 | 181.781 | 303.470 | 4.73E+00 | 2999.0 | 75.39% |
|  | SSO | 190.607 | 181.779 | 317.995 | 1.99E+01 | 3146.7 | 75.29% |
| S | CGS | **42852455.318** | **42852371.414** | **42852558.603** | **5.13E+01** | 73.7 | 53.05% |
|  | iSSO | 42884123.930 | 42855796.800 | 42962706.304 | 2.31E+04 | **83.1** | **53.17%** |
|  | MLS | 42852542.658 | 42852379.638 | 42852760.659 | 1.07E+02 | 74.6 | 53.08% |
|  | PSO | 43086777.786 | 42895417.355 | 43164499.230 | 1.71E+06 | 75.6 | 53.08% |
|  | SSO | 48694736.491 | 42917355.151 | 51068015.101 | 2.55E+06 | 80.4 | 53.10% |
| W | CGS | 5388293.921 | 5388254.053 | 5388305.721 | 1.74E+01 | 354.8 | 62.07% |
|  | iSSO | **5388248.279** | **5388248.279** | **5388248.279** | **4.69E-08** | **1776.9** | 62.15% |
|  | MLS | 5388287.251 | 5388255.804 | 5388304.274 | 1.73E+01 | 1444.6 | **62.16%** |
|  | PSO | 5402677.843 | 5416819.727 | 5775435.394 | 2.20E+05 | 1562.0 | 61.81% |
|  | SSO | 5615666.191 | 5418746.297 | 6334283.992 | 2.24E+05 | 1596.6 | 61.77% |
| Y | CGS | 123.030 | 122.621 | 123.307 | 1.55E-01 | 30.0 | 55.15% |
|  | iSSO | **121.676** | **121.666** | **121.692** | **7.54E-03** | **223.6** | **55.76%** |
|  | MLS | 122.765 | 122.259 | 123.024 | 1.70E-01 | 175.4 | 55.38% |
|  | PSO | 122.614 | 121.925 | 123.192 | 2.41E-01 | 188.4 | 55.47% |
|  | SSO | 122.502 | 121.825 | 123.657 | 4.43E-01 | 212.7 | 55.60% |

#### **Table A3.** Experimental results for *T*=.1 and *p*=2.5.

| ID | Alg. | F_avg_ | F_min_ | F_max_ | F_std_ | N_avg_ | F_measure_ |
| --- | --- | --- | --- | --- | --- | --- | --- |
| A | CGS | 1119.877 | 1001.375 | 1194.126 | 5.18E+01 | 30.0 | 56.13% |
|  | iSSO | **971.528** | 949.852 | **994.403** | **1.13E+01** | 51.8 | **59.37%** |
|  | MLS | 1113.767 | 1003.281 | 1189.605 | 5.11E+01 | **52.6** | 56.08% |
|  | PSO | 1021.244 | 983.659 | 1063.073 | 3.47E+01 | 52.2 | 57.28% |
|  | SSO | 982.976 | **949.219** | 1031.477 | 1.80E+01 | 52.1 | 59.34% |
| B | CGS | 425.041 | 425.017 | 425.083 | **2.11E-02** | 123.4 | 96.07% |
|  | iSSO | **424.886** | **424.867** | **424.972** | 2.66E-02 | **356.6** | **96.16%** |
|  | MLS | 425.048 | 425.019 | 425.109 | 2.31E-02 | 318.0 | 96.07% |
|  | PSO | 536.298 | 429.327 | 710.511 | 1.04E+01 | 345.4 | 95.14% |
|  | SSO | 643.169 | 429.760 | 920.421 | 1.62E+02 | 349.7 | 95.11% |
| C | CGS | 7102.042 | 7084.435 | 7111.887 | 6.59E+00 | 41.8 | 39.05% |
|  | iSSO | **7069.794** | **7069.146** | **7070.432** | **3.35E-01** | **150.6** | **39.33%** |
|  | MLS | 7101.748 | 7090.556 | 7109.392 | 4.62E+00 | 133.4 | 39.06% |
|  | PSO | 7112.362 | 7083.804 | 7111.005 | 6.81E+00 | 145.4 | 39.06% |
|  | SSO | 7118.424 | 7083.733 | 7158.897 | 2.31E+01 | 145.9 | 39.08% |
| G | CGS | 1086.044 | 1065.056 | 1108.036 | 1.36E+01 | 73.3 | 41.01% |
|  | iSSO | **1059.336** | **1059.336** | **1059.337** | **6.21E-06** | **1780.4** | **41.39%** |
|  | MLS | 1066.982 | 1061.802 | 1073.640 | 2.46E+00 | 1464.2 | 41.21% |
|  | PSO | 1125.075 | 1061.612 | 1184.909 | 3.94E+01 | 1566.3 | 41.19% |
|  | SSO | 1133.027 | 1059.623 | 1239.264 | 4.40E+01 | 1672.5 | 41.26% |
| I | CGS | 185.392 | 181.993 | 189.498 | 2.10E+00 | 52.3 | 75.06% |
|  | iSSO | **181.728** | **181.728** | **181.728** | **6.70E-08** | **3316.6** | 75.26% |
|  | MLS | 182.092 | 181.852 | 182.530 | 1.81E-01 | 2944.7 | 75.17% |
|  | PSO | 187.867 | 181.806 | 185.324 | 4.18E+00 | 3132.6 | **75.26%** |
|  | SSO | 188.486 | 181.797 | 218.370 | 7.43E+00 | 3144.9 | 75.26% |
| S | CGS | **42852459.008** | 42852374.682 | **42852546.837** | **4.77E+01** | 73.6 | 53.08% |
|  | iSSO | 42907267.124 | 42856063.373 | 43263206.325 | 7.65E+04 | **83.5** | 53.12% |
|  | MLS | 42852530.876 | **42852372.572** | 42852727.014 | 9.14E+01 | 73.6 | 53.10% |
|  | PSO | 43278899.643 | 42909905.482 | 43922657.684 | 1.51E+06 | 74.4 | **53.12%** |
|  | SSO | 48096798.949 | 42913396.696 | 51075445.642 | 2.90E+06 | 80.4 | 53.09% |
| W | CGS | 5388291.089 | 5388253.695 | 5388306.884 | 1.83E+01 | 355.1 | 62.06% |
|  | iSSO | **5388248.279** | **5388248.279** | **5388248.279** | **6.11E-08** | **1779.7** | 62.08% |
|  | MLS | 5388284.054 | 5388253.278 | 5388304.250 | 1.76E+01 | 1441.9 | **62.14%** |
|  | PSO | 5590744.291 | 5394757.526 | 5424692.405 | 3.20E+04 | 1589.6 | 62.10% |
|  | SSO | 5620979.821 | 5394825.337 | 6505178.716 | 2.70E+05 | 1595.7 | 62.03% |
| Y | CGS | 123.064 | 122.712 | 123.304 | 1.52E-01 | 30.0 | 55.11% |
|  | iSSO | **121.677** | **121.662** | **121.691** | **7.23E-03** | **223.2** | **55.70%** |
|  | MLS | 122.729 | 122.221 | 123.007 | 1.58E-01 | 174.8 | 55.42% |
|  | PSO | 122.669 | 121.861 | 123.717 | 5.21E-01 | 191.4 | 55.57% |
|  | SSO | 122.616 | 121.821 | 123.766 | 5.69E-01 | 212.0 | 55.57% |

#### **Table A4.** Experimental results for *T*=.3 and *p*=1.5.

| ID | Alg. | F_avg_ | F_min_ | F_max_ | F_std_ | N_avg_ | F_measure_ |
| --- | --- | --- | --- | --- | --- | --- | --- |
| A | CGS | 1123.012 | 1009.075 | 1228.471 | 5.94E+01 | 30.0 | 56.02% |
|  | iSSO | **951.533** | 948.779 | **955.146** | **1.84E+00** | **152.9** | **59.74%** |
|  | MLS | 1070.164 | 1003.839 | 1136.559 | 3.26E+01 | 138.6 | 56.41% |
|  | PSO | 1020.810 | 979.853 | 1139.957 | 5.23E+01 | 142.6 | 57.74% |
|  | SSO | 1014.424 | **948.355** | 1148.939 | 5.52E+01 | 146.6 | 59.72% |
| B | CGS | 425.032 | 425.017 | 425.059 | 1.10E-02 | 128.6 | 96.03% |
|  | iSSO | **424.867** | **424.867** | **424.867** | **1.71E-05** | **1023.2** | **96.12%** |
|  | MLS | 425.026 | 425.017 | 425.041 | 6.57E-03 | 908.6 | 96.05% |
|  | PSO | 462.811 | 428.794 | 427.989 | 6.60E+01 | 927.8 | 95.20% |
|  | SSO | 688.806 | 429.630 | 920.421 | 1.52E+02 | 1000.1 | 95.00% |
| C | CGS | 7105.222 | 7089.053 | 7115.084 | 5.76E+00 | 42.3 | 39.10% |
|  | iSSO | **7068.630** | **7068.628** | **7068.632** | **1.08E-03** | **436.2** | **39.25%** |
|  | MLS | 7097.357 | 7087.497 | 7106.186 | 4.85E+00 | 381.3 | 39.12% |
|  | PSO | 7097.977 | 7088.131 | 7224.576 | 8.35E+00 | 405.1 | 39.24% |
|  | SSO | 7139.556 | 7090.143 | 7270.067 | 3.00E+01 | 418.8 | 39.13% |
| G | CGS | 1079.456 | 1065.447 | 1100.008 | 1.09E+01 | 74.5 | 41.03% |
|  | iSSO | **1059.336** | **1059.336** | **1059.336** | **7.59E-09** | **5099.8** | **41.42%** |
|  | MLS | 1064.821 | 1061.748 | 1066.972 | 1.11E+00 | 4189.2 | 41.23% |
|  | PSO | 1137.653 | 1070.790 | 1203.109 | 1.93E+01 | 4636.3 | 40.90% |
|  | SSO | 1140.545 | 1088.868 | 1239.791 | 3.44E+01 | 4767.2 | 40.18% |
| I | CGS | 185.018 | 181.989 | 188.817 | 1.71E+00 | 54.1 | 75.04% |
|  | iSSO | **181.728** | **181.728** | **181.728** | **2.04E-11** | **9494.0** | **75.31%** |
|  | MLS | 181.973 | 181.814 | 182.179 | 9.48E-02 | 8426.1 | 75.12% |
|  | PSO | 184.558 | 181.813 | 194.394 | 9.61E+00 | 8646.0 | 75.23% |
|  | SSO | 193.185 | 181.809 | 229.209 | 1.34E+01 | 8904.6 | 75.26% |
| S | CGS | **42852411.899** | 42852370.453 | **42852470.585** | **2.80E+01** | 184.1 | 53.00% |
|  | iSSO | 42852441.557 | **42852356.230** | 42852682.740 | 7.27E+01 | **247.9** | **53.07%** |
|  | MLS | 42852455.306 | 42852372.709 | 42852568.693 | 4.47E+01 | 185.3 | 53.07% |
|  | PSO | 43689275.604 | 42908405.571 | 48895496.168 | 1.42E+06 | 207.6 | 53.01% |
|  | SSO | 48690700.425 | 43190487.293 | 51077400.563 | 2.39E+06 | 229.0 | 52.70% |
| W | CGS | 5388291.824 | 5388255.337 | 5388306.806 | 1.70E+01 | 352.8 | **62.09%** |
|  | iSSO | **5388248.279** | **5388248.279** | **5388248.279** | **4.70E-09** | **5095.6** | 62.07% |
|  | MLS | 5388267.671 | 5388252.890 | 5388300.864 | 1.25E+01 | 4122.7 | 62.03% |
|  | PSO | 5597254.329 | 5392744.957 | 6063180.519 | 3.07E+04 | 4404.3 | 62.01% |
|  | SSO | 5618586.300 | 5407015.806 | 6322497.323 | 2.20E+05 | 4565.7 | 61.87% |
| Y | CGS | 123.046 | 122.649 | 123.330 | 1.69E-01 | 30.0 | 55.11% |
|  | iSSO | **121.661** | **121.660** | **121.664** | **1.26E-03** | **646.9** | **55.68%** |
|  | MLS | 122.605 | 122.264 | 122.828 | 1.33E-01 | 502.5 | 55.30% |
|  | PSO | 122.696 | 121.951 | 123.119 | 3.22E-01 | 597.4 | 55.55% |
|  | SSO | 122.805 | 121.821 | 123.744 | 5.59E-01 | 606.9 | 55.57% |

#### **Table A5.** Experimental results for *T*=.3 and *p*=2.0.

| ID | Alg. | F_avg_ | F_min_ | F_max_ | F_std_ | N_avg_ | F_measure_ |
| --- | --- | --- | --- | --- | --- | --- | --- |
| A | CGS | 756.097 | 604.321 | 882.431 | 6.98E+01 | 30.0 | 56.10% |
|  | iSSO | **590.591** | **590.531** | **590.698** | **4.36E-02** | **216.3** | 57.43% |
|  | MLS | 676.111 | 613.411 | 727.105 | 2.94E+01 | 175.4 | 55.35% |
|  | PSO | 630.985 | 593.797 | 722.281 | 2.54E+01 | 195.5 | 57.16% |
|  | SSO | 609.864 | 591.219 | 644.418 | 1.62E+01 | 201.2 | **57.46%** |
| B | CGS | 1154.199 | 1154.144 | 1154.284 | 3.42E-02 | 119.7 | 96.02% |
|  | iSSO | 1529.169 | **1153.963** | 3498.999 | 8.68E+02 | **1432.7** | **96.16%** |
|  | MLS | **1154.157** | 1154.133 | **1154.189** | **1.59E-02** | 1214.5 | 96.11% |
|  | PSO | 1312.007 | 1154.943 | 1614.692 | 4.83E+02 | 1371.2 | 96.07% |
|  | SSO | 2081.956 | 1155.027 | 3498.999 | 8.14E+02 | 1422.2 | 96.09% |
| C | CGS | 11568.915 | 11509.364 | 11592.457 | 1.52E+01 | 35.6 | 39.10% |
|  | iSSO | **11472.011** | **11472.001** | **11472.035** | **8.51E-03** | **624.4** | **39.32%** |
|  | MLS | 11538.887 | 11500.306 | 11553.681 | 1.22E+01 | 500.5 | 39.15% |
|  | PSO | 11588.332 | 11493.476 | 11608.030 | 2.36E+01 | 506.0 | 39.31% |
|  | SSO | 11594.629 | 11492.678 | 11640.568 | 2.68E+01 | 589.0 | 39.20% |
| G | CGS | 1080.006 | 1063.941 | 1102.569 | 1.24E+01 | 73.3 | 41.13% |
|  | iSSO | **1059.336** | **1059.336** | **1059.336** | **6.87E-09** | **5100.1** | **41.33%** |
|  | MLS | 1064.327 | 1062.107 | 1066.085 | 8.66E-01 | 4189.6 | 41.21% |
|  | PSO | 1104.265 | 1063.253 | 1186.407 | 1.62E+01 | 4654.7 | 41.18% |
|  | SSO | 1130.237 | 1067.553 | 1198.979 | 2.73E+01 | 4786.3 | 41.12% |
| I | CGS | 185.327 | 181.826 | 190.076 | 2.55E+00 | 54.3 | 75.14% |
|  | iSSO | **181.728** | **181.728** | **181.728** | **1.83E-11** | **9495.6** | **75.36%** |
|  | MLS | 181.987 | 181.804 | 182.177 | 9.77E-02 | 8427.6 | 75.15% |
|  | PSO | 186.633 | 181.806 | 206.307 | 5.66E+00 | 8599.4 | 75.15% |
|  | SSO | 186.753 | 181.824 | 210.650 | 5.75E+00 | 8889.6 | 75.17% |
| S | CGS | **42852405.328** | 42852368.882 | **42852456.610** | **2.33E+01** | 184.1 | 53.14% |
|  | iSSO | 42852454.042 | **42852348.975** | 42852724.919 | 9.39E+01 | **248.0** | 53.19% |
|  | MLS | 42852443.362 | 42852369.231 | 42852540.947 | 4.80E+01 | 186.6 | 53.21% |
|  | PSO | 42997199.117 | 42873766.291 | 45988538.000 | 4.60E+04 | 221.9 | **53.25%** |
|  | SSO | 48714622.911 | 42976247.001 | 51306693.890 | 1.99E+06 | 229.6 | 53.08% |
| W | CGS | 5388289.998 | 5388253.354 | 5388304.342 | 1.70E+01 | 351.4 | 62.14% |
|  | iSSO | **5388248.279** | **5388248.279** | **5388248.279** | **4.70E-09** | **5095.7** | 62.22% |
|  | MLS | 5388267.508 | 5388251.649 | 5388299.217 | 1.22E+01 | 4121.0 | **62.24%** |
|  | PSO | 5434548.662 | 5393460.828 | 5869908.173 | 1.44E+05 | 4148.7 | 62.15% |
|  | SSO | 5594093.117 | 5394069.543 | 6158230.348 | 1.84E+05 | 4565.4 | 62.22% |
| Y | CGS | 123.074 | 122.331 | 123.375 | 1.91E-01 | 30.0 | 55.03% |
|  | iSSO | **121.661** | **121.660** | **121.663** | **8.33E-04** | **646.2** | **55.36%** |
|  | MLS | 122.611 | 122.284 | 122.818 | 1.28E-01 | 501.4 | 55.13% |
|  | PSO | 122.649 | 122.266 | 123.894 | 4.88E-01 | 558.4 | 55.16% |
|  | SSO | 122.705 | 121.783 | 123.969 | 6.06E-01 | 607.1 | 55.31% |

#### **Table A6.** Experimental results for *T*=.3 and *p*=2.5.

| ID | Alg. | F_avg_ | F_min_ | F_max_ | F_std_ | N_avg_ | F_measure_ |
| --- | --- | --- | --- | --- | --- | --- | --- |
| A | CGS | 479.037 | 393.871 | 552.339 | 4.36E+01 | 32.7 | 56.08% |
|  | iSSO | **377.129** | **377.097** | **377.226** | **3.42E-02** | **152.3** | 58.64% |
|  | MLS | 439.281 | 382.666 | 497.725 | 3.08E+01 | 142.1 | 57.74% |
|  | PSO | 380.669 | 380.430 | 428.003 | 2.31E+01 | 145.8 | 58.11% |
|  | SSO | 380.591 | 377.116 | 403.228 | 5.83E+00 | 146.3 | **58.67%** |
| B | CGS | 3150.141 | 3149.501 | 3150.823 | 2.83E-01 | 108.8 | 96.04% |
|  | iSSO | **3149.307** | **3149.307** | **3149.307** | **6.53E-05** | **1007.4** | 96.17% |
|  | MLS | 3149.798 | 3149.520 | 3149.980 | 1.38E-01 | 910.0 | 96.09% |
|  | PSO | 5774.377 | 3149.760 | 11037.004 | 2.23E+03 | 941.9 | **96.17%** |
|  | SSO | 6617.849 | 3149.859 | 14252.203 | 4.40E+03 | 1003.3 | 96.06% |
| C | CGS | 18882.673 | 18779.542 | 18950.959 | 3.85E+01 | 31.9 | 39.03% |
|  | iSSO | **18701.041** | **18700.112** | **18701.936** | **4.71E-01** | **436.9** | **39.28%** |
|  | MLS | 18829.661 | 18767.878 | 18870.041 | 2.21E+01 | 382.7 | 39.13% |
|  | PSO | 18838.015 | 18755.120 | 18916.918 | 4.49E+01 | 387.4 | 39.16% |
|  | SSO | 18862.156 | 18741.648 | 18940.843 | 5.46E+01 | 419.5 | 39.18% |
| G | CGS | 1844.445 | 1841.744 | 1845.645 | 8.58E-01 | 91.9 | 41.01% |
|  | iSSO | **1839.825** | **1839.825** | **1839.825** | **4.98E-12** | **3418.5** | **41.20%** |
|  | MLS | 1842.662 | 1840.787 | 1843.911 | 7.74E-01 | 3028.9 | 41.08% |
|  | PSO | 1956.564 | 1840.808 | 1853.464 | 5.35E+01 | 3093.5 | 41.05% |
|  | SSO | 2068.512 | 1840.815 | 2197.251 | 1.03E+02 | 3249.7 | 41.09% |
| I | CGS | 184.509 | 183.345 | 186.837 | 8.51E-01 | 54.5 | 75.14% |
|  | iSSO | **183.037** | **183.037** | **183.037** | **1.46E-11** | **5828.8** | **75.45%** |
|  | MLS | 183.338 | 183.133 | 183.560 | 1.27E-01 | 5474.3 | 75.23% |
|  | PSO | 183.921 | 183.095 | 190.461 | 3.51E+00 | 5574.5 | 75.29% |
|  | SSO | 186.176 | 183.038 | 195.443 | 3.60E+00 | 5627.3 | 75.35% |
| S | CGS | 715669601.039 | 715669072.691 | **715670030.105** | **2.38E+02** | 159.1 | 53.08% |
|  | iSSO | **715669229.900** | **715668527.249** | 715671566.427 | 6.87E+02 | **201.2** | **53.27%** |
|  | MLS | 715669881.793 | 715668876.682 | 715670689.895 | 3.81E+02 | 160.7 | 53.09% |
|  | PSO | 759925304.209 | 715738772.497 | 763831106.504 | 5.46E+05 | 183.5 | 53.13% |
|  | SSO | 802177646.252 | 715928759.955 | 874556763.480 | 4.75E+07 | 189.4 | 53.17% |
| W | CGS | 75840729.808 | 75840302.962 | 75840812.271 | 1.24E+02 | 368.2 | 62.03% |
|  | iSSO | **75840193.934** | **75840193.934** | **75840193.934** | **1.49E-08** | **3660.7** | 62.15% |
|  | MLS | 75840580.592 | 75840243.362 | 75840776.845 | 1.67E+02 | 3171.6 | 62.13% |
|  | PSO | 76173897.116 | 75842209.368 | 103593357.982 | 5.49E+06 | 3199.4 | **62.15%** |
|  | SSO | 78929406.717 | 75844285.293 | 117757772.518 | 7.81E+06 | 3390.4 | 62.04% |
| Y | CGS | 75.158 | 74.140 | 75.692 | 3.58E-01 | 30.0 | 55.00% |
|  | iSSO | **72.837** | **72.833** | **72.857** | **5.41E-03** | **465.7** | **56.15%** |
|  | MLS | 74.491 | 73.582 | 74.813 | 2.78E-01 | 380.5 | 55.46% |
|  | PSO | 74.343 | 73.185 | 75.418 | 6.40E-01 | 399.8 | 55.84% |
|  | SSO | 74.094 | 72.952 | 75.856 | 8.53E-01 | 445.4 | 56.05% |

#### **Table A7.** Experimental results for *T*=.5 and *p*=1.5.

| ID | Alg. | F_avg_ | F_min_ | F_max_ | F_std_ | N_avg_ | F_measure_ |
| --- | --- | --- | --- | --- | --- | --- | --- |
| A | CGS | 1131.622 | 1043.620 | 1210.991 | 4.18E+01 | 30.0 | 56.03% |
|  | iSSO | **948.807** | **948.218** | **949.613** | **4.01E-01** | **254.8** | **61.82%** |
|  | MLS | 1049.454 | 975.125 | 1100.530 | 3.00E+01 | 226.9 | 60.03% |
|  | PSO | 1046.733 | 966.564 | 1173.640 | 4.91E+01 | 237.5 | 60.54% |
|  | SSO | 1032.788 | 948.831 | 1182.669 | 6.71E+01 | 242.7 | 61.66% |
| B | CGS | 425.038 | 425.017 | 425.088 | 1.83E-02 | 122.8 | 96.03% |
|  | iSSO | **424.867** | **424.867** | **424.867** | **1.12E-06** | **1685.5** | **96.15%** |
|  | MLS | 425.023 | 425.016 | 425.033 | 4.14E-03 | 1498.7 | 96.04% |
|  | PSO | 526.800 | 425.952 | 541.836 | 1.34E+02 | 1598.8 | 95.90% |
|  | SSO | 720.732 | 427.980 | 920.421 | 1.47E+02 | 1650.1 | 95.47% |
| C | CGS | 7103.898 | 7091.877 | 7113.753 | 5.79E+00 | 42.1 | 39.02% |
|  | iSSO | **7068.628** | **7068.628** | **7068.628** | **8.33E-06** | **722.9** | **39.28%** |
|  | MLS | 7094.258 | 7086.475 | 7101.098 | 3.65E+00 | 629.7 | 39.15% |
|  | PSO | 7094.850 | 7078.800 | 7133.042 | 1.62E+01 | 638.3 | 39.22% |
|  | SSO | 7122.917 | 7076.143 | 7157.560 | 1.98E+01 | 689.3 | 39.13% |
| G | CGS | 1078.469 | 1064.420 | 1104.610 | 1.19E+01 | 73.4 | 41.08% |
|  | iSSO | **1059.336** | **1059.336** | **1059.336** | **3.77E-11** | **8413.4** | **41.38%** |
|  | MLS | 1063.945 | 1062.096 | 1065.160 | 6.94E-01 | 6908.3 | 41.20% |
|  | PSO | 1084.164 | 1064.520 | 1173.049 | 6.85E+00 | 7620.2 | 41.13% |
|  | SSO | 1136.735 | 1067.919 | 1258.334 | 4.02E+01 | 7874.3 | 40.95% |
| I | CGS | 185.963 | 182.375 | 191.631 | 2.58E+00 | 52.5 | 75.05% |
|  | iSSO | **181.728** | **181.728** | **181.728** | **2.01E-13** | **15672.1** | **75.40%** |
|  | MLS | 181.958 | 181.822 | 182.113 | 8.18E-02 | 13901.7 | 75.31% |
|  | PSO | 182.997 | 182.091 | 183.046 | 3.73E+00 | 14655.0 | 75.30% |
|  | SSO | 188.356 | 182.094 | 211.999 | 6.74E+00 | 14815.2 | 75.28% |
| S | CGS | 42852397.249 | 42852370.685 | 42852433.916 | 1.79E+01 | 264.3 | 53.08% |
|  | iSSO | **42852347.416** | **42852345.652** | **42852350.697** | **1.51E+00** | **412.4** | 53.18% |
|  | MLS | 42852433.190 | 42852369.956 | 42852519.659 | 4.30E+01 | 316.0 | **53.18%** |
|  | PSO | 44768109.975 | 43081143.123 | 44101688.208 | 5.67E+05 | 375.9 | 52.83% |
|  | SSO | 49357703.836 | 44059122.887 | 51235091.088 | 1.84E+06 | 377.8 | 51.71% |
| W | CGS | 5388295.119 | 5388253.775 | 5388306.467 | 1.64E+01 | 357.4 | 62.02% |
|  | iSSO | **5388248.279** | **5388248.279** | **5388248.279** | **4.70E-09** | **8408.2** | **62.16%** |
|  | MLS | 5388262.279 | 5388252.207 | 5388281.346 | 7.51E+00 | 6808.5 | 62.08% |
|  | PSO | 5474386.337 | 5391587.819 | 5898029.366 | 1.52E+05 | 6912.9 | 62.10% |
|  | SSO | 5588309.668 | 5392742.540 | 6289291.248 | 1.95E+05 | 7531.0 | 62.00% |
| Y | CGS | 123.057 | 122.309 | 123.333 | 1.86E-01 | 30.0 | 55.02% |
|  | iSSO | **121.660** | **121.660** | **121.661** | **3.28E-04** | **1070.2** | **55.48%** |
|  | MLS | 122.567 | 122.103 | 122.773 | 1.32E-01 | 826.8 | 55.16% |
|  | PSO | 122.678 | 122.035 | 123.241 | 3.98E-01 | 935.9 | 55.20% |
|  | SSO | 122.766 | 121.797 | 123.875 | 5.67E-01 | 1002.2 | 55.37% |

#### **Table A8.** Experimental results for *T*=.5 and *p*=2.0.

| ID | Alg. | F_avg_ | F_min_ | F_max_ | F_std_ | N_avg_ | F_measure_ |
| --- | --- | --- | --- | --- | --- | --- | --- |
| A | CGS | 748.113 | 627.928 | 842.198 | 5.92E+01 | 30.0 | 56.04% |
|  | iSSO | **590.537** | **590.528** | **590.555** | **7.85E-03** | **360.7** | 59.59% |
|  | MLS | 663.411 | 620.262 | 702.660 | 2.22E+01 | 292.0 | 56.83% |
|  | PSO | 661.505 | 615.455 | 732.714 | 2.57E+01 | 314.5 | 57.25% |
|  | SSO | 610.968 | 590.606 | 756.603 | 2.66E+01 | 330.9 | **59.72%** |
| B | CGS | 1154.208 | 1154.143 | 1154.300 | 3.82E-02 | 121.4 | 96.06% |
|  | iSSO | 1388.467 | **1153.963** | 3498.999 | 7.11E+02 | **2368.2** | **96.17%** |
|  | MLS | **1154.151** | 1154.133 | **1154.186** | **1.16E-02** | 2001.8 | 96.15% |
|  | PSO | 2231.231 | 1154.270 | 2713.858 | 8.35E+02 | 2249.5 | 96.16% |
|  | SSO | 2580.784 | 1155.027 | 3498.999 | 9.94E+02 | 2347.7 | 96.09% |
| C | CGS | 11560.565 | 11528.987 | 11578.003 | 1.25E+01 | 35.9 | 39.12% |
|  | iSSO | **11472.001** | **11472.000** | **11472.003** | **6.98E-04** | **1032.5** | **39.38%** |
|  | MLS | 11535.208 | 11503.226 | 11550.755 | 1.22E+01 | 824.8 | 39.31% |
|  | PSO | 11542.208 | 11501.482 | 11588.655 | 2.21E+01 | 900.8 | 39.32% |
|  | SSO | 11582.015 | 11493.703 | 11630.487 | 3.60E+01 | 971.2 | 39.26% |
| G | CGS | 1081.607 | 1063.832 | 1103.550 | 1.14E+01 | 74.3 | 41.02% |
|  | iSSO | **1059.336** | **1059.336** | **1059.336** | **1.77E-11** | **8419.7** | **41.22%** |
|  | MLS | 1064.227 | 1062.868 | 1065.330 | 6.86E-01 | 6917.5 | 41.16% |
|  | PSO | 1139.354 | 1068.693 | 1083.595 | 2.20E+01 | 7187.9 | 40.99% |
|  | SSO | 1143.170 | 1094.749 | 1231.280 | 3.29E+01 | 7838.4 | 39.88% |
| I | CGS | 185.548 | 182.222 | 190.287 | 2.26E+00 | 53.4 | 75.00% |
|  | iSSO | **181.728** | **181.728** | **181.728** | **2.01E-13** | **15669.6** | 75.30% |
|  | MLS | 181.931 | 181.802 | 182.070 | 7.51E-02 | 13899.3 | 75.27% |
|  | PSO | 185.561 | 181.797 | 276.626 | 2.16E+01 | 14610.7 | **75.32%** |
|  | SSO | 194.892 | 181.795 | 295.792 | 2.33E+01 | 14624.4 | 75.30% |
| S | CGS | 42852397.750 | 42852371.388 | 42852429.247 | 1.49E+01 | 265.4 | 53.01% |
|  | iSSO | **42852347.460** | **42852345.678** | **42852351.912** | **1.61E+00** | **412.8** | **53.10%** |
|  | MLS | 42852422.554 | 42852374.589 | 42852499.813 | 3.67E+01 | 314.9 | 53.05% |
|  | PSO | 44278040.069 | 42920193.229 | 44307766.908 | 3.93E+05 | 370.5 | 53.04% |
|  | SSO | 49027227.071 | 43427702.266 | 51196207.012 | 2.17E+06 | 378.6 | 52.32% |
| W | CGS | 5388293.129 | 5388253.174 | 5388305.886 | 1.63E+01 | 346.1 | 62.11% |
|  | iSSO | **5388248.279** | **5388248.279** | **5388248.279** | **4.70E-09** | **8406.8** | **62.28%** |
|  | MLS | 5388261.829 | 5388253.935 | 5388281.262 | 6.65E+00 | 6798.8 | 62.16% |
|  | PSO | 5503168.184 | 5393914.158 | 5784027.335 | 7.79E+04 | 7177.5 | 62.13% |
|  | SSO | 5598251.365 | 5419159.319 | 6220838.446 | 1.88E+05 | 7527.7 | 61.76% |
| Y | CGS | 123.119 | 122.806 | 123.365 | 1.56E-01 | 30.0 | 55.03% |
|  | iSSO | **121.660** | **121.660** | **121.660** | **1.82E-04** | **1070.1** | 55.55% |
|  | MLS | 122.572 | 122.212 | 122.777 | 1.48E-01 | 827.4 | 55.31% |
|  | PSO | 122.805 | 122.191 | 123.202 | 1.83E-01 | 905.9 | 55.39% |
|  | SSO | 122.846 | 121.780 | 124.270 | 6.86E-01 | 1002.2 | **55.57%** |

#### **Table A9.** Experimental results for *T*=.5 and *p*=2.5.

| ID | Alg. | F_avg_ | F_min_ | F_max_ | F_std_ | N_avg_ | F_measure_ |
| --- | --- | --- | --- | --- | --- | --- | --- |
| A | CGS | 469.554 | 387.472 | 560.261 | 4.56E+01 | 32.7 | 56.03% |
|  | iSSO | **377.100** | **377.096** | **377.115** | **3.92E-03** | **254.3** | **57.70%** |
|  | MLS | 421.565 | 380.618 | 466.982 | 2.18E+01 | 228.1 | 57.05% |
|  | PSO | 418.457 | 377.634 | 426.647 | 1.96E+01 | 238.2 | 57.59% |
|  | SSO | 385.472 | 377.157 | 414.058 | 1.04E+01 | 241.3 | 57.69% |
| B | CGS | 3150.117 | 3149.576 | 3150.691 | 2.79E-01 | 109.3 | 96.13% |
|  | iSSO | **3149.307** | **3149.307** | **3149.307** | **6.13E-06** | **1664.2** | **96.29%** |
|  | MLS | 3149.753 | 3149.495 | 3149.938 | 1.33E-01 | 1503.0 | 96.15% |
|  | PSO | 4481.502 | 3149.506 | 13338.357 | 8.09E+02 | 1601.8 | 96.26% |
|  | SSO | 5437.025 | 3149.746 | 14252.203 | 3.07E+03 | 1653.2 | 96.23% |
| C | CGS | 18886.520 | 18810.995 | 18939.135 | 2.93E+01 | 31.9 | 39.14% |
|  | iSSO | **18700.092** | **18699.980** | **18700.198** | **5.83E-02** | **722.6** | **39.51%** |
|  | MLS | 18822.646 | 18758.233 | 18846.196 | 1.91E+01 | 631.0 | 39.29% |
|  | PSO | 18865.702 | 18756.418 | 18847.437 | 2.37E+01 | 687.0 | 39.27% |
|  | SSO | 18882.613 | 18745.636 | 18961.255 | 5.44E+01 | 691.8 | 39.31% |
| G | CGS | 1844.671 | 1842.189 | 1847.295 | 9.08E-01 | 89.2 | 41.04% |
|  | iSSO | **1839.825** | **1839.825** | **1839.825** | **0.00E+00** | **5640.1** | 41.13% |
|  | MLS | 1842.592 | 1840.919 | 1843.844 | 7.05E-01 | 4995.7 | **41.13%** |
|  | PSO | 1891.350 | 1845.708 | 2051.637 | 1.16E+01 | 5127.6 | 41.07% |
|  | SSO | 2073.455 | 1853.978 | 2220.152 | 8.66E+01 | 5362.1 | 40.84% |
| I | CGS | 184.700 | 183.256 | 187.220 | 9.98E-01 | 55.0 | 75.13% |
|  | iSSO | **183.037** | **183.037** | **183.037** | **3.17E-13** | **9631.7** | 75.33% |
|  | MLS | 183.234 | 183.097 | 183.460 | 9.00E-02 | 9034.1 | 75.22% |
|  | PSO | 187.444 | 183.071 | 233.743 | 1.68E+01 | 9116.7 | 75.33% |
|  | SSO | 192.803 | 183.038 | 378.287 | 2.87E+01 | 9281.6 | **75.36%** |
| S | CGS | 715669491.572 | 715668936.907 | 715669797.235 | 2.06E+02 | 218.8 | 53.13% |
|  | iSSO | **715668522.370** | **715668514.306** | **715668539.019** | **6.51E+00** | **334.2** | **53.27%** |
|  | MLS | 715669683.548 | 715669046.802 | 715670068.813 | 2.51E+02 | 272.9 | 53.18% |
|  | PSO | 746505767.342 | 717013876.367 | 790105710.651 | 3.04E+07 | 276.9 | 53.15% |
|  | SSO | 813788271.908 | 717020575.449 | 870810468.425 | 4.64E+07 | 311.6 | 53.08% |
| W | CGS | 75840711.477 | 75840204.873 | 75840808.679 | 1.52E+02 | 369.2 | 62.00% |
|  | iSSO | **75840193.934** | **75840193.934** | **75840193.934** | **4.21E-08** | **6048.0** | **62.15%** |
|  | MLS | 75840440.820 | 75840215.865 | 75840748.967 | 1.69E+02 | 5232.4 | 62.00% |
|  | PSO | 76219094.786 | 75840661.402 | 75893975.767 | 1.60E+05 | 5564.7 | 62.12% |
|  | SSO | 77157046.429 | 75843307.919 | 80776460.731 | 1.23E+06 | 5593.6 | 62.09% |
| Y | CGS | 75.233 | 74.178 | 75.608 | 3.03E-01 | 30.0 | 55.10% |
|  | iSSO | **72.833** | **72.833** | **72.836** | **9.49E-04** | **769.6** | **56.21%** |
|  | MLS | 74.435 | 74.093 | 74.821 | 1.68E-01 | 627.2 | 55.21% |
|  | PSO | 73.982 | 74.072 | 74.902 | 3.74E-01 | 675.7 | 55.19% |
|  | SSO | 73.942 | 73.038 | 75.630 | 6.96E-01 | 734.4 | 56.09% |
